# LAPAROSCOPIC ANTIREFLUX SURGERY: ARE OLD QUESTIONS ANSWERED? USEFUL FOR EXTRA-ESOPHAGEAL SYMPTOMS?

**DOI:** 10.1590/0102-672020210002e1632

**Published:** 2022-01-31

**Authors:** Marco G. PATTI, Fernando Augusto Mardiros HERBELLA

**Affiliations:** 1 Department of Medicine and Surgery, University of North Carolina at Chapel Hill, Chapel Hill, NC, USA; 2 Departmento de Cirurgia, Escola Paulista de Medicina, Universidade Federal de São Paulo - UNIFESP, Sao Paulo, Brasil

**Keywords:** Gastroesophageal Reflux, Idiopathic Pulmonary Fibrosis, Manometry, Fundoplication, Doença do refluxo gastroesofágico, Fibrose pulmonar idiopática, Manometria, Fundoplicatura

## Abstract

Gastroesophageal reflux disease is usually associated with esophageal or typical symptoms such as heartburn, regurgitation, and dysphagia. However, there is today mounting evidence that gastroesophageal reflux can also cause extra-esophageal or atypical problems such as cough, aspiration pneumonia, and pulmonary fibrosis. AIM: The aim of this study was to discuss the pathophysiology of extra-esophageal symptoms, the diagnostic evaluation, complications, and the outcome of video laparoscopic antireflux surgery.

**METHODS:**

This study analyzes the recent literature review.

**RESULTS:**

It is important to separate patients with respiratory symptoms into two different groups: *group I*: patients having typical symptoms such as heartburn and respiratory symptoms, and *group II*: patients having respiratory symptoms only, in whom reflux is otherwise silent.

**CONCLUSIONS:**

Gastroesophageal reflux can cause respiratory symptoms in addition to esophageal typical symptoms. High index of suspicion should be present, and a complete workup was done to diagnose whether pathologic reflux is present and whether it extends to the proximal esophagus or pharynx. Antireflux surgery in these patients should be considered, as it is safe and effective.

## RESUMO - RACIONAL

A doença do refluxo gastroesofágico geralmente está associada a sintomas esofágicos ou típicos, como azia, regurgitação e disfagia. No entanto, existem hoje evidências crescentes, que o refluxo gastroesofágico também pode causar problemas extraesofágicos ou atípicos, como tosse, pneumonia por aspiração e fibrose pulmonar.

**OBJETIVO:**

discutir a fisiopatologia dos sintomas extraesofágicos, avaliação diagnóstica, complicações e o resultado da cirurgia videolaparoscópica antirrefluxo.

**MÉTODOS:**

Análise de revisão recente da literatura. RESULTADOS: É importante separar os pacientes com sintomas respiratórios em dois grupos distintos: grupo I: pacientes que apresentam sintomas típicos como azia e sintomas respiratórios e grupo II: pacientes que apresentam apenas sintomas respiratórios, nos quais o refluxo é silencioso.

**CONCLUSÕES:**

O refluxo gastroesofágico pode causar sintomas respiratórios além dos sintomas esofágicos típicos. Elevado índice de suspeita deve estar presente e uma avaliação completa deve ser feita para diagnosticar se o refluxo patológico está presente e se ele se estende ao esôfago proximal ou faringe. A cirurgia anti-refluxo nesses pacientes deve ser considerada, pois é segura e eficaz.

## INTRODUCTION

Gastroesophageal reflux disease (GERD) is usually associated with esophageal or typical symptoms such as heartburn, regurgitation, and dysphagia. However, there is today mounting evidence that gastroesophageal reflux can also cause extra-esophageal or atypical problems, particularly respiratory complications such as cough, and aspiration pneumonia. Recent evidence also suggests that GERD might be implicated-either alone or in combination with other factors-in the genesis of idiopathic pulmonary fibrosis (IPF).

The key for successful treatment is a clear understanding of the pathophysiology of reflux, a thorough workup, and the execution of an effective fundoplication.

## METHODS

### Pathophysiology

There are two accepted theories to explain how reflux can cause respiratory symptoms: the reflex theory and the reflux theory.

According to the *reflex theo*ry, esophageal mucosa stimulation can lead to triggering of the vagus nerves. The vagal stimulation in turn would cause bronchoconstriction and patients would experience cough or asthma.

According to the *reflux theory*, acid can reach the upper esophagus, and eventually microaspiration can cause respiratory symptoms and lung damage.

In 1993, we first described *High Gastroesophageal Reflux* (H-GER) and characterized its clinical and functional aspects^13^. After esophageal manometry, patients underwent ambulatory pH monitoring, using a special catheter with two antimony sensors located 5 and 20 cm above the upper border of the manometrically determined lower esophageal sphincter (LES).

*Clinicall*y, laryngitis, pulmonary aspiration, and hoarseness were present in patients with H-GER. Interestingly, heartburn was not present in many of these patients.

*Pathophysiologically*, these patients had often a pan-esophageal motor disorder involving the LES, the peristalsis, and the upper esophageal sphincter (UES). The LES was weaker and shorter, the amplitude of peristalsis was lower in both the distal and proximal esophagus with a higher incidence of simultaneous waves, and the UES resting pressure was lower.

Sweet et al. studied that 109 patients awaited for lung transplantation by esophageal manometry and pH monitoring^19^ and found that patients with pathologic reflux had a high prevalence of a hypotensive LES (55%) and impaired esophageal peristalsis (47%). Distal reflux was present in 68% of patients, and proximal reflux was present in 37% of patients, suggesting the possibility of microaspiration.

It is known that reflux determines mucosal damage, from esophagitis to Barrett’s esophagus, therefore creating a vicious cycle as it can promote ineffective esophageal motility (IEM) and worsen esophageal acid exposure. Diener et al. reviewed the findings of esophageal manometry among 1006 consecutive patients with pathologic reflux as shown in ambulatory pH monitoring^5^. Peristalsis was normal in 56% of patients, IEM (amplitude of peristalsis <30 mmHg or >30% simultaneous waves) was present in 21%, and a nonspecific esophageal motility disorder was present in 25% of patients. Compared with the two other groups, patients with IEM had more severe reflux, slower acid clearance, worse mucosal injury, and more frequent respiratory symptoms.

Other factors play a role in the pathogenesis of GERD. Schlottmann et al. showed that the presence and size of a hiatal hernia determine weaker peristalsis and worse acid reflux and that patients with a hiatal hernia > 5 cm more often experience respiratory symptoms^18^. Obesity also plays an important role as it determines an increase in the transdiaphragmatic pressure gradient, which may overcome the resistance posed by the LES^3^. Herbella et al. showed that in case of patients with morbid obesity for every 5-point increase in the body mass index, there was a 3-point increase in the reflux score^7^.

Finally, it is very important from a therapeutic point of view to remember that both acid and bile are present in the gastric refluxate but the medications available today can only decrease or block the acid component so that they only affect the pH of the refluxate (from acidic to weakly acidic or alkaline), but not the total number of reflux episodes^14^.

### Diagnostic workup

A complete workup is necessary for the diagnosis of GERD^2^ ^,^ ^15^. Symptoms and endoscopy (in the absence of Barrett’s esophagus), in fact, have been shown to have low sensitivity and specificity. For instance, Patti et al. studied that by esophageal manometry and pH monitoring (esophageal function tests [EFTs]), 822 patients thought to have GERD based on the symptomatic evaluation and endoscopic assessment (patients with biopsy-proven Barrett’s esophagus were excluded)^15^. Notably, 30% of patients (247 patients) were found to have a normal esophageal acid exposure (GERD negative). This study emphasized that (1) symptoms were unreliable in the diagnosis of GERD; (2) low-grade esophagitis (grades I and II) was diagnostically nonspecific; and (3) only pH monitoring identified patients with GERD. Similarly, Bello et al. performed EFT in 136 patients referred for antireflux surgery^2^. After excluding two patients who were discovered to have achalasia, pathologic reflux was found in only 78 (58%) patients.

In 2013, an Esophageal Diagnostic Advisory Panel composed by both gastroenterologists and surgeons clearly defined the ideal preoperative workup before antireflux surgery^9^. It was recognized that a *barium swallow* is not useful for the diagnosis of GERD but rather to define the anatomy (i.e., presence, size, and type of hiatal hernia and stricture). An *upper endoscopy* is important to rule out other pathologies such as gastritis or eosinophilic esophagitis and to determine the presence and degree of mucosal injury. However, 50-60% of patients with pathologic reflux shown by pH monitoring do not have esophagitis. Only the presence of biopsy-proven Barrett’s esophagus is diagnostic of GERD.

*Esophageal manometry* is not diagnostic, but it is important to rule out a primary motility disorder, such as achalasia, to determine the position of the LES for the pH monitoring and to characterize peristalsis, allowing the choice of the proper antireflux operation^4^ ^,^ ^12^.

*Ambulatory pH monitoring* (off medications) is of key importance. This test defines whether pathologic reflux is present and whether the symptoms experienced by the patients are due to reflux. The temporal correlation between symptoms and episodes of reflux can be established by either the symptom index or the symptom-associated probability. This test is particularly important in patients with respiratory symptoms as they often have silent reflux and do not experience heartburn. *Esophageal pH monitoring* can be combined with impedance to detect reflux independently from the pH (acidic, weakly acidic, alkaline)^8^.

The definitive proof that aspiration of gastric contents is occurring is provided by the determination of pepsin in the bronchoalveolar lavage fluid^6^. As pepsin (pepsinogen is released by the gastric chief cells and converted to pepsin by the hydrochloric acid released by the parietal cells) is normally absent in the esophagus and trachea, it is a very sensitive marker for aspiration.

## RESULTS

It is important to separate patients with respiratory symptoms into two different groups: *group I*, patients having typical symptoms such as heartburn and respiratory symptoms, and *group II*, patients having respiratory symptoms only, in whom reflux is otherwise silent.

## DISCUSSION

### Antireflux surgery

*Group I.* Laparoscopic fundoplication controls heartburn and regurgitation in about 90% of patients, but the effect on the respiratory symptoms is less predictable. The uncertainty stems from the difficulty to determine preoperatively whether cough or wheezing is caused by reflux when reflux is shown by pH monitoring. Many studies have shown that EFTs are of key importance.

Patti et al. studied the effect of laparoscopic fundoplication on GERD-induced respiratory symptoms^16^. Each patient was studied preoperatively by esophageal manometry and a dual-probe pH monitoring and the correlation between cough and episodes of reflux in the lower and upper esophagus was established (cough was considered due to reflux when it occurred during or within 3 min from an episode of reflux). Overall, heartburn resolved in 91% of patients, regurgitation in 90% of patients, cough in 74% of patients, and wheezing in 64% of patients. Interestingly, cough resolved in 57% of patients when no temporal correlation was found, in 77% of patients when a correlation was found between cough and reflux in the distal esophagus, and in 90% of patients when cough correlated with reflux in the distal and proximal esophagus. These results clearly illustrated the value of pH monitoring in establishing a correlation between cough and reflux and in predicting the outcome of therapy.

Hoppo et al. studied the effect of antireflux surgery in patients with chronic cough and abnormal proximal exposure as measured by hypopharyngeal multichannel intraluminal impedance in 49 patients with chronic cough^8^. Abnormal proximal acid exposure was discovered in 36 (73%) of 49 patients. At a median follow-up of 4.6 months, 13 (81%) of 16 patients who underwent antireflux surgery had resolution of cough, and 3 (19%) patients had a significant improvement. The authors concluded that hypopharyngeal multichannel intraluminal impedance improves the sensitivity of laryngopharyngeal reflux diagnosis and helps predict which patients will respond to antireflux surgery. These results have been confirmed by other studies and clearly show that once a cause-and-effect relationship between reflux and respiratory symptoms has been established, antireflux surgery should be the primary form of treatment.

*Group II.* In the study of patients awaiting lung transplantation, Sweet et al. found that symptoms such as heartburn, regurgitation, or dysphagia did not distinguish patients with and without reflux and that about one-third of patients eventually found to have reflux were asymptomatic^19^. As there is evidence that GERD plays a role, either alone or in combination with other factors, in the genesis of IPF, every patient with this diagnosis, regardless of symptoms, should be screened with EFTs and treatment should be started if the patients are found to have pathologic reflux^1^ ^,^ ^10^ ^,^ ^11^. As discussed, the gastric refluxate is a mixture of acid and bile and the current acid-reducing medications just change the pH of the refluxate but do not block reflux as the number of episodes is unchanged. Based on these considerations, and until medications that can restore the competence of the gastroesophageal junction are available, antireflux surgery should be the primary form of treatment for patients with IPF who can have general anesthesia.

A study from the University of California San Francisco and the Mayo Clinic by Lee et al. showed that GERD therapy was associated with longer survival in patients with IPF^10^. Specifically, they showed that acid-reducing medications were associated with a lower radiologic fibrosis score and longer survival as compared with patients not taking medications and that the longest survival was seen in patients after a Nissen fundoplication. These findings clearly supported the hypothesis that GERD and microaspiration may play a role in the pathogenesis of IPF.

Based on these data, a multicenter, randomized, controlled trial was started in six academic centers in the United States in June 2014^17^. Over the following 2 years, 58 patients with IPF were randomized to either a no-surgery group (29 patients) or a surgery group (29 patients). The primary end point was the evaluation of the forced vital capacity (FVC) at 48 months, which was done in 20 patients in no-surgery group and 27 patients in surgery group, respectively. The results showed that there was no effect on the FVC by treatment, but acute exacerbations, respiratory-related hospitalizations, and death were less common in the surgery group.

Overall, antireflux surgery was safe and well tolerated. Even though the primary end point of change in FVC failed to reach statistical significance, it is noted that this study was severely underpowered as 400 patients, rather than 58, were required to achieve 90% power.

## CONCLUSIONS

Gastroesophageal reflux can cause respiratory symptoms in addition to more typical symptoms. As symptoms such as heartburn and regurgitation have low sensitivity and specificity for the diagnosis of GERD and reflux may be silent in many patients, a high index of suspicion should be present, and a complete workup was done to diagnose whether pathologic reflux is present and whether it extends to the proximal esophagus or pharynx. Therefore, antireflux surgery should be considered in these patients as it is safe and effective.
